# Searching for a prognostic index in lupus nephritis

**DOI:** 10.1186/s40001-022-00946-y

**Published:** 2023-01-11

**Authors:** E. Rodríguez-Almaraz, E. Gutiérrez-Solís, E. Rabadán, P. Rodríguez, M. Alonso, L. Carmona, M. J. García de Yébenes, E. Morales, M. Galindo-Izquierdo

**Affiliations:** 1 Department of Rheumatology, University Hospital “12 de Octubre”, Avda. Córdoba Km 5.400, 28041 Madrid, Spain; 2 Department of Nephrology, University Hospital “12 de Octubre”, Madrid, Spain; 3 Department of Pathology, University Hospital “12 de Octubre”, Madrid, Spain; 4 Instituto de Salud Musculoesquelética (Inmusc), Madrid, Spain; 5 Research Institute of University Hospital “12 de Octubre” (imas12), Madrid, Spain; 6 Department of Medicine, Complutense University, Madrid, Spain

**Keywords:** Lupus nephritis, Prognostic index, Poor renal evolution

## Abstract

**Background:**

Currently we do not have an ideal biomarker in lupus nephritis (LN) that should help us to identify those patients with SLE at risk of developing LN or to determine those patients at risk of renal progression. We aimed to evaluate the development of a prognostic index for LN, through the evaluation of clinical, analytical and histological factors used in a cohort of lupus. We have proposed to determine which factors, 6 months after the diagnosis of LN, could help us to define which patients will have a worse evolution of the disease and may be, more aggressive treatment and closer follow-up.

**Methods:**

A retrospective study to identify prognostic factors was carried out. We have included patients over 18 years of age with a clinical diagnosis of systemic lupus erythematosus (SLE) and kidney involvement confirmed by biopsy, who are followed up in our centre during the last 20 years. A multi-step statistical approach will be used in order to obtain a limited set of parameters, optimally selected and weighted, that show a satisfactory ability to discriminate between patients with different levels of prognosis.

**Results:**

We analysed 92 patients with LN, although only 73 have been able to be classified according to whether or not they have presented poor renal evolution. The age of onset (44 vs. 32; *p* = 0.024), the value of serum creatinine (1.41 vs. 1.04; *p* = 0.041), greater frequency of thrombocytopenia (30 vs. 7%; *p* = 0.038), higher score in the renal chronicity index (2.47 vs. 1.04; *p* = 0.015), proliferative histological type (100%) and higher frequency of interstitial fibrosis (67 vs. 32%; *p* = 0.017) and tubular atrophy (67 vs. 32%; *p* = 0.018) was observed between two groups. The multivariate analysis allowed us to select the best predictive model for poor outcome at 6 months based on different adjustment and discrimination parameters.

**Conclusion:**

We have developed a prognostic index of poor renal evolution in patients with LN that combines demographic, clinical, analytical and histopathological factors, easy to use in routine clinical practice and that could be an effective tool in the early detection and management.

## Key messages

1. Development a prognostic index of poor renal evolution in patients with LN.
2. We use clinical, histological and laboratory factors 6 months after diagnosis and treatment
3. Effective tool in the early detection and management, easy to use in clinical practice

## Introduction

Lupus nephritis (LN) is one of the most common manifestations of systemic lupus erythematosus (SLE), affecting approximately 40% of patients with lupus. It represents a major risk factor for morbidity and mortality, and 10% of patients with LN will develop end-stage kidney disease (ESKD) [1, 2].

The survival of patients with SLE has improved in recent decades. This improvement is due to advances in the diagnosis and treatment [3]. Despite this improvement, we currently lack good biomarkers to predict the course of lupus nephritis, the best therapeutic option or the response to treatment. Remission is achieved in 20–30% of the patients within 6–12 months from the onset of LN and 20%–35% of those patients relapse within 3–5 years. At least, 20% of LN patients develop chronic kidney disease (CKD) and 5–20% reach ESKD within 10 years from the LN onset. The management of immunosuppression utilized in LN requires highly nuanced care [4]. This reinforces the importance of early detection and treatment when looking for adequate long-term outcomes. In this way, Ayoub et al. [5] tried to develop a prediction model of treatment response in LN after 12 months of diagnosis. Early decrease in proteinuria predicts good long-term renal outcome, however, while the positive predictive value of this target was excellent, the negative predictive value was poor.

Our group have recently published a systematic review about the potential prognostic factors in LN. The main contributing factors have been serum creatinine (SCr), glomerular filtration rate (eGFR), levels of C3, C1q and anti-DNA antibodies. The histological factors that marked the evolution of renal function were class IV and V, interstitial and vascular involvement, and the chronicity index [6].

Nowadays, we do not have adequate biomarkers in clinical practice to predict the prognosis of patients with lupus nephritis. For this reason, the aim of this study was the development of a prognostic index for LN through the evaluation of clinical, analytical and histological factors used in a cohort of lupus patients in our hospital. This prognostic index should be easy to apply to routine clinical practice and be able to select those patients who would require closer monitoring to prevent the development of CKD.

## Methods

This retrospective study was carried out at University Hospital “12 de Octubre”, a 1,200-bed tertiary care centre in Madrid, Spain. We selected patients ≥ 18 years diagnosed with SLE (regardless of vital status), according to the 1997 American College of Rheumatology (ACR) revised criteria [7] and kidney involvement confirmed by biopsy according to International Society of Nephrology/Renal Pathology Society (ISN/RPS) classification [8], who are followed up in our centre during the last 20 years. The institution’s Ethical and Research Committee approved the study (approval number: 17/061), including the current analysis. Participants gave informed consent to participate in the study before taking part.

### Variables and measurements

Data collection were done from clinical charts and we obtained information from the following domains: (1) demographics; (2) chronological; (3) general clinical data, including vital status; (4) cumulative manifestations of SLE, defined by the glossaries of the ACR criteria for classification of SLE and an activity index, SLE Disease Activity Index (SLEDAI); (5) comorbidities, including cardiovascular risk factors and cause of death; and (6) treatments previous of LN and induction and maintenance therapy for LN. Antiphospholipid syndrome was defined according to the Sydney criteria [9].

The main variable was poor renal evolution and was defined by the presence of at least one of the following:

- *Non-response to treatment* Active urine sediment, proteinuria > 0.5 g/d, impaired renal function (eGFR < 90 ml/min or deterioration > 10% compared to baseline filtration if it was altered, calculated with the estimation of glomerular filtration rate of Chronic Kidney Disease Epidemiology Collaboration (CKD-EPI) [10]).
- *Recurrences of kidney involvement* Understanding recurrence as the increase in the activity of the disease that requires intensifying treatment. We defined relapse as reappearance or significant increase in haematuria (> 15 red cells/field) with dysmorphic red cells and/or casts and/or sustained increase in proteinuria (≥ 1 g/24 h or ≥ 1 g/g in patients with complete remission or ≥ 50% of baseline proteinuria in patients with partial remission) and/or a decrease in eGFR ≥ 25% not attributable to other causes [11].
- *Renal failure* Defined according to Systemic Lupus International Collaborating Centers (SLICC) criteria [12] for chronic renal damage as creatinine clearance (estimated/measured) < 50%, proteinuria ≥ 3.5 g/24 h or end-stage renal disease (regardless of dialysis or kidney transplant) maintained for 6 months.

As independent variables, all the potential prognostic factors, as well as the possible confounding factors and the usual descriptive variables, were collected from the clinical history. The following independent variables were used:

- Demographics: age at onset of nephritis, gender, and ethnicity.
- Cardiovascular risk factors prior to nephritis.
- Lupus activity: extrarenal manifestations and baseline SLEDAI [13].
- Serological activity: anti-dsDNA antibodies by IFI; antiphospholipid profile (lupus anticoagulant (LA) positive (based on aPPT, silica test or dRVVT) or anticardiolipin (ACL) IgG and/or IgM or—antiB2glycoprotein (aB2GP1) IgG or IgM) > 40 UFL/ml; low C3 (< 83 mg/dl); low C4 (< 14 mg/dl).
- Analytical data of kidney involvement: SCr, eGFR, 24-h proteinuria, uPCR, haematuria.
- Histological data: activity index, chronicity index, histological type, interstitial fibrosis, tubular atrophy and thrombotic microangiopathy (TMA). Pathologic lesions were evaluated according to the International Society of Nephrology and the Renal Pathology Society (ISN/RPS) systems Austin system of semiquantitative scores for activity and chronicity was applied (Table 1) [14].

**Table 1:** Scores for activity and chronicity (Austin system)

Activity index (0–24)	
Endocapillary hypercellularity	(0–3)
Leukocytic infiltration	(0–3)
Subendothelial hyaline deposits	(0–3)
Fibrinoid necrosis/caryorrhexis	(0–3) × 2
Epithelial crescents	(0–3) × 2
Interstitial inflammation	(0–3)
Chronicity index (0–12)	
Glomerular sclerosis	(0–3)
Fibrous crescents	(0–3)
Tubular atrophy	(0–3)
Interstitial fibrosis	(0–3)

### Statistic analysis

A multi-step statistical approach will be used in order to obtain a limited set of parameters, optimally selected and weighted, that show a satisfactory ability to discriminate between patients with different levels of prognosis.

Continuous variables were tested for normality to decide which type of hypothesis tests to use. The only one that presented normal distribution was glomerular filtration rate, and in this case Student's *t*-test was used. The rest of the continuous variables did not present normality criteria, so the Mann–Whitney *U* test was used.

#### Creation of the dependent variable “poor renal evolution”

A combined variable will be constructed in which poor renal evolution at 6 months, defined by the existence of at least one of the following situations:

- Recurrence of kidney involvement.
- Chronic kidney disease presence.
- Need for dialysis or transplant.
- Lack of response to treatment.

#### Description of the analysis sample and comparison of patients with and without poor renal evolution

A descriptive study of the baseline situation of the patients will be carried out, both globally and by both groups. For the description, measures of central tendency and dispersion will be used, as well as tables of frequencies and distribution of percentages for quantitative and qualitative variables, respectively. For the comparison of the groups with and without poor renal evolution, parametric or non-parametric hypothesis contrast tests will be used depending on the distribution of the variables.

##### Bivariate analysis

The association between prognostic factors and poor renal outcome will be studied using bivariate logistic regression models using poor renal outcome as the dependent variable and the prognostic factors described in the literature and defined by the panel of experts as independent variables.

##### Multivariate analysis

The predictive model will be estimated using multivariate logistic regression models, introducing into the model the prognostic factors with theoretical meaning and those that present a p value of less than 0.250 in the bivariate analysis. Successive models will be built until reaching the most parsimonious and with the lowest Akaike and Bayesian information criteria (AIC and BIC). The discrimination power of the model will be quantified by the area under the ROC curve of the final logistic model. Discriminatory power is defined as the model's ability to correctly classify subjects according to whether or not they have poor renal outcomes.

## Results

### Baseline characteristics

The sample has 92 patients with LN, although only 73 have been able to be classified according to whether or not they have presented poor renal evolution due to missing data. The majority are women (82%), of Caucasian ethnicity (70%) and a mean age at the onset of LN of 34 ± 15 years. The patients present mean SLEDAI values of 16 ± 7; SCr 1.12 ± 0.8 mg/dl; eGFR 84.3 ± 4.7 ml/min/1.73m^2^, proteinuria 3.51 ± 3.45 g/24 h and mean values in the indices of renal activity and chronicity of 4.56 ± 3.84 and 1.34 ± 1.59, respectively. 75% of patients have extrarenal manifestations, and 11% thrombocytopenia. The most frequent histological types (78%) are the proliferative forms (types III or IV or a combination with type V). Most patients do not have interstitial fibrosis (62%) or tubular atrophy (66%). From a serological point of view, 76% had anti-DNA antibodies, 29% anticardiolipin antibodies, and 20% lupus anticoagulant. In addition, there are low values of complement C3 and C4 in 67% and 64% of cases, respectively. Finally, the most used prior treatment was steroids (60%).

A description of the total sample at baseline was made and the baseline status of the groups with and without poor renal progression at 6 months was compared (Table 2).

**Table 2:** Baseline characteristics: total and by renal evolution at 6 months

Characteristics	Total (*n* = 73)	No CKD progression (*n* = 58)	CKD progression (*n* = 15)	*P* value
Continuos variables: average ± SD				
Age of onset (years)	34.4 ± 15.5	32.2 ± 13.9	44.4 ± 19.1	0.024*
SLEDAI	16.1 ± 7.3	16.7 ± 7.7	13.9 ± 5.2	0.179
Creatinine (mg/dl)	1.12 ± 0.81	1.04 ± 0.76	1.41 ± 0.94	0.041*
Glomerular filtrate rate (ml/min/1.73m^2^)	84.3 ± 4.7	88.3 ± 5.2	69.8 ± 9.8	0.106
Proteinuria (g/24 h)	3.51 ± 3.45	3.83 ± 3.76	2.35 ± 1.55	0.155
Renal activity index	4.56 ± 3.84	4.58 ± 4.09	4.47 ± 2.75	0.804
Chronicity activity index	1.34 ± 1.59	1.05 ± 1.26	2.47 ± 2.20	0.015*
Categorical variables: n (%)				
Woman	60 (82.2%)	50 (86.2%)	10 (66.7%)	0.078
Ethnicity				
Caucasian	48 (70.6%)	38 (70.4%)	10 (71.4%)	
Asian	2 (2.9%)	2 (3.7%)	–	
Arab	2 (2.9%)	2 (3.7%)	–	
Hispanic	14 (20.6%)	11 (20.4%)	3 (21.4%)	
Others	2 (2.9%)	1 (1.8%)	1 (7.1%)	
Extrarenal manifestations	53 (75.7%)	41 (73.2%)	12 (85.7%)	0.492
Haemolytic anaemia	7 (10.3%)	5 (9.1%)	2 (15.4%)	0.611
CNS diffuse involvement	7 (10.0%)	7 (12.5%)	–	1.331
Thrombocytopenia (< 50.000)	8 (11.8%)	4 (7.3%)	4 (30.7%)	0.038*
Haematuria	56 (80.0%)	43 (78.2%)	13 (86.7%)	0.718
Cell casts	32 (49.2%)	27 (50.0%)	5 (45.4%)	0.783
Histology type				0.031*
No proliferative (mesangial and membranous)	16 (22.2%)	16 (28.1%)	–	
Proliferative	56 (77.8%)	41 (71.9%)	15 (100%)	
Anti-DNA antibodies	52 (76.5%)	41 (75.9%)	11 (78.6%)	1.000
Lupus anticoagulant	10 (20.0%)	8 (18.6%)	2 (28.6%)	0.616
Anticardiolipins (Ig G or Ig M)	15 (29.4%)	12 (29.7%)	3 (30.0%)	1.000
Anti β2 glycoproteins (Ig G/Ig M)	2 (7.7%)	2 (9.5%)	–	1.000
Low C3	47 (67.1%)	40 (72.7%)	7 (46.7%)	0.057
Low C4	45 (64.3%)	36 (65.4%)	9 (60.0%)	0.696
Interstitial fibrosis				
No	45 (61.6%)	40 (69.0%)	5 (33.3%)	
Yes	28 (38.4%	18 (31.0%)	10 (66.7%)	
Tubular atrophy				
No	44 (66.1%)	39 (68.4%)	5 (33.3%)	
Yes	28 (38.9%)	18 (31.6%)	10 (66.7%)	
Thrombotic microangiopathy	–	–	–	
Previous antimalarial treatment	22 (32.8%)	19 (34.5%)	3 (25.0%)	0.737
Previous glucocorticoids treatment	42 (60.9%)	36 (64.3%)	6 (46.1%)	0.228
Previous immunosuppressor treatment	18 (26.1%)	14 (25.0%)	4 (30.8%)	0.670

*SLEDAI* Systemic Lupus Erythematosus Disease Activity Index, *CNS* central nervous system; *: statistically significant

### Evolution of patients depending on poor renal outcomes

The main differences between the two groups at six months were age of onset (44 vs. 32; *p* = 0.024), SCr higher values (1.41 vs. 1.04; *p* = 0.041), higher score in the renal chronicity index (2.47 vs. 1.04; *p* = 0.015), greater frequency of thrombocytopenia (30 vs. 7%; *p* = 0.038), proliferative histological type (100%) and higher frequency of interstitial fibrosis (67 vs. 32%; *p* = 0.017) and tubular atrophy (67 vs. 32%; *p* = 0.018) (Table 1).

The results of the bivariate analysis showed that the factors that increase the probability of poor renal evolution at 6 months are the patient's age (OR = 1.05; *p* = 0.020), the highest score in the renal chronicity index (OR = 1.67; *p* = 0.006), the presence of interstitial fibrosis (OR = 4.44; *p* = 0.016) or tubular atrophy (OR = 4.33; *p* = 0.018) and the thrombocytopenia platelets < 50,000 per mm3) (OR = 5.67; *p* = 0.029) (Table 3).

**Table 3:** Predictors of poor renal evolution at 6 months

Predictor	Bivariate OR [CI 95%]	*P* value
Age of onset (years)	**1.05 [1.01–1.09]**	0.020**
Time to kidney biopsy (months)	1.00 [0.99–1.01]	0.496
Woman (%)	0.32 [0.09–1.18]	0.088
SLEDAI	0.94 [0.86–1.03]	0.192
Creatinine mg/dl	1.61 [0.85–3.03]	0.140
Glomerular filtrate rate (ml/in/1.73m^2^)	0.98 [0.97–1.00]	0.110
Proteinuria (g/24 h)	0.80 [0.59–1.08]	0.147
Renal chronicity index	**1.67 [1.16–2.42]**	0.006 **
Extrarenal manifestations	2.19 [0.44–11.0]	0.338
Thrombocytopenia*	**5.67 [1.19–26.9]**	0.029 **
Low C3	0.33 [0.10–1.06]	0.063
Previous antimalarial treatment	0.48 [0.14–1.61]	0.233
Previous glucocorticoids treatment	0.63 [0.15–2.61]	0.526
Previous immunosuppressor treatment	1.33 [0.35–5.01]	0.670
Interstitial fibrosis	**4.44 [1.33–14.9]**	0.016 **
Tubular atrophy	**4.33 [1.29–14.5]**	0.018 **

*SLEDAI* Systemic Lupus Erythematosus Disease Activity Index; **: statistically significant

* < 50.000 platelets/mm^3 ^

**Fig. 1:**
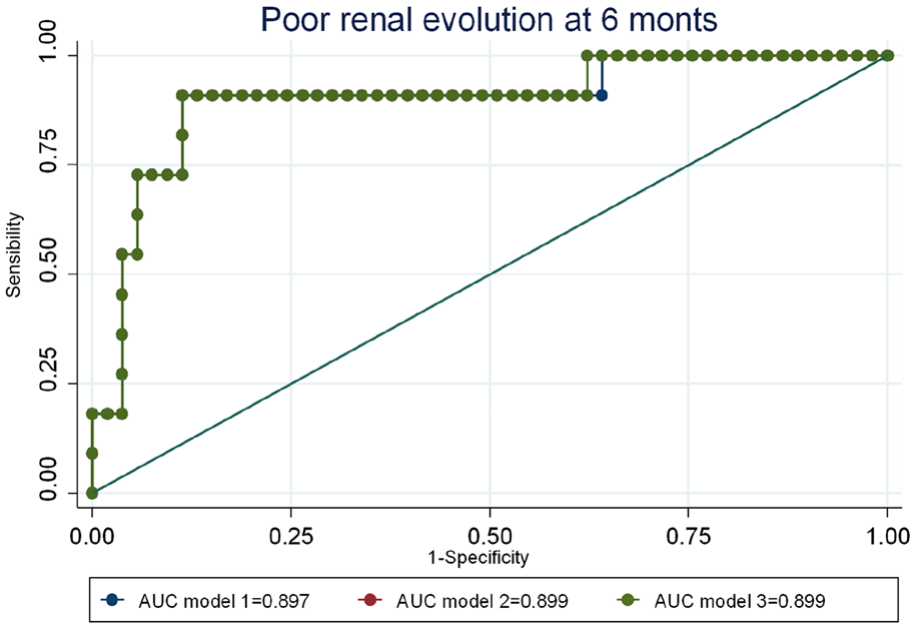
Comparison of ROC curves of the selected models: 6 months

The multivariate analysis allowed us to select the 3 best predictive models for poor outcome at 6 months based on different adjustment and discrimination parameters (Table 4). The areas under the ROC curve (AUC) obtained in this model, ranged between 0.897 and 0.899, with no statistically significant differences (Fig. 1). The predicted probability cut-off point was chosen by the model that maximized the values of sensitivity (correct classification of poor outcomes), specificity (correct classification of negatives) and the percentage of global classification. The main predictors of poor renal evolution were thrombocytopenia, with OR greater than 30, and interstitial fibrosis, with OR greater than 20, although in both cases the confidence intervals were very wide (Table 4).

**Table 4:** Predictive models of poor evolution at 6 months

Variables	Model 1 OR [CI 95%]	Model 1 *p* value	Model 2 OR [CI 95%]	Model 2 *p* value	Model 3 OR [CI 95%]	Model 3 *p* value
Age (years)	1.04 [0.98–1.11]	0.180	1.04 [0.98–1.11]	0.180	1.04 [0.98–1.11]	0.178
SLEDAI	0.99 [0.85–1.15]	0.922				
SCr (mg/dl)	0.91 [0.13–6.10]	0.921	0.91 [0.13–6.09]	0.922		
Proteinuria (g/24 h)	0.83 [0.49–1.38]	0.467	0.82 [0.51–1.31]	0.405	0.81 [0.51–1.28]	0.377
Chronicity index	0.76 [0.26–2.23]	0.614	0.75 [0.26–2.17]	0.595	0.72 [0.31–1.67]	0.453
Thrombocytopenia*	**22.0 [1.07–452.5]**	**0.045**	**21.7 [1.1–428.1]**	**0.043**	**20.3 [1.43–287.4]**	0.026 **
Fibrosis	**33.6 [1.31–861.4]**	**0.034**	**34.8 [1.45–833.1]**	**0.028**	**35.9 [1.57–820.8]**	0.025 **
Glucocorticoids	0.32 [0.05–2.01]	0.233	0.32 [0.05–2.07]	0.232	0.32 [0.05–2.06]	0.230
Low C3	0.48 [0.04–5.14]	0.543	0.45 [0.05–3.98]	0.477	0.45 [0.05–3.94]	0.473
Constant	0.038 [0–3.83]	0.165	0.036 [0–3.25]	0.148	0.03 [0–3.17]	0.145
HL		0.156		0.218		0.306
AUC	0.897 (0.780–1.000)		0.899 (0.785–1.000)		0.899 (0.785–1.000)	
Best cut point	Pr predicted ≥ 0.243 S = 90.9% E = 88.7% %CC = 89.1		Pr predicted ≥ 0.235 S = 90.9% E = 88.7% %CC = 89,1		Pr predicted ≥ 0.225 S = 90.9% E = 88.7% %CC = 89.1	

*SLEDAI* Systemic Lupus Erythematosus Disease Activity Index, *Scr.* serum creatinine, *HL* Hosmer–Lemeshow goodness-of-fit test, *Pr* probability, *S* sensitivity, *E* specificity, *CC* correct classification; **: statistically significant

* < 50.000 platelets/mm^3^

The model chosen for poor outcome at 6 months is shown in Table 5. In equality of sensitivity and specificity, we have chosen the model with the highest predicted probability. We have analysed prognosis factors of poor outcomes in LN at 12 months, but this will be discussed in another paper.

**Table 5:** Predictive model of poor evolution at 6 months

Variables	Model OR	Model *p* value
Age (years)	1.04 [0.98–1.11]	0.180
SLEDAI	0.99 [0.85–1.15]	0.922
SCr (mg/dl)	0.91 [0.13–6.10]	0.921
Proteinuria (g/24 h)	0.83 [0.49–1.38]	0.467
Chronicity index	0.76 [0.26–2.23]	0.614
Thrombocytopenia*	**22.0 [1.07–452.5]**	0.045 **
Fibrosis	**33.6 [1.31–861.4]**	0.034 **
Glucocorticoids	0.32 [0.05–2.01]	0.233
Low C3	0.48 [0.04–5.14]	0.543
Constant	0.038 [0–3.83]	0.165
HL		0.156
AUC	0.897 (0.780–1.000)	
Best cut point	Pr predicted ≥ 0.243 *S* = 90.9% *E* = 88.7% % CC = 89.1	

*SLEDAI *Systemic Lupus Erythematosus Disease Activity Index,* Scr. *serum creatinine, *HL* Hosmer–Lemeshow goodness-of-fit test, *Pr* probability, *S* sensitivity, *E* specificity; *CC* correct classification; **: statistically significant

* < 50.000 platelets/mm^3^

## Discussion

In this retrospective study in patients with LN, we have designed a prognostic index for evolution of renal function in patients with lupus nephritis. The main predictors of poor renal evolution were thrombocytopenia and interstitial fibrosis. Our findings highlight the value of thrombocytopenia and histology to determine renal survival in patients with LN.

In our study, older patients (44.4 ± 19.1 years) had worst evolution renal function compared with younger patients (32.2 ± 13.9 years). Kang et al. [15] found similar results in 117 patients with LN followed during follow-up during a mean of 76.5 months. They divided them into three groups based on age: juvenile LN (JLN) if < 8 years old, adult LN (ALN) between 18 and 50 years old and late-onset LN (LLN) if > 50 years old. The study findings showed that the patients with LLN had a higher chronicity index, developed CKD and death higher than JLN and ALN patients.

Several studies have shown that tubular atrophy and interstitial fibrosis were independent factors for poor renal evolution [16–23] as well as the chronicity index [24]. Tang et al. [25] have developed and validated a risk score for the development of ESRD in LN, emphasizing the importance of tubulointerstitial lesions (tubular atrophy and interstitial fibrosis) than the histological subtype according to the ISN/RPS classification [26]. These renal histopathological changes will be considered a chronic change and loss of function of the nephrons and therefore they are related to the poor renal evolution. Ayoub et al. [5] have developed a prediction model of treatment in LN, showing that early detection and treatment of NL was essential to achieve good long-term renal outcomes. In this predictive model they have used classical biomarkers (proteinuria, renal glomerular filtration rate) and new urinary biomarkers (cytokines, chemokines). This study has showed that the predictive value of proteinuria in LN is complicated because proteinuria may represent acute kidney injury due to inflammation and podocyte dysfunction, or chronic kidney injury due to scarring after inflammation. However, clinical and demographic variables were relatively more important than any novel urine biomarker.

A recent systematic review by our group on the main prognostic factors in the outcome of CKD has shown that the classical biomarkers (proteinuria, GFR and urinary sediment) remain despite advances in the diagnosis and treatment of lupus nephritis [6]. One of the main limitations of clinical trials in LN has been considering renal function and proteinuria as the only criteria for assessing response to treatment. However, the concept of a histopathological target emerged from observations that clinical outcome based on proteinuria and/or urinalysis and histopathological outcome based on repeat kidney biopsies are discordant. Recent studies have shown that an activity and chronicity index > 3 correlates with a higher incidence of relapse and CKD, respectively, in lupus nephritis [27]. The nuances of histological lesions have become a cornerstone of the evolution of renal function. Several publications have shown that chronic damage in the tubulointerstitial compartment and different kinds of vascular lesions contributed significantly to the association with poor long-term renal function [21–23, 28, 29]. Korbet et al. [18] have showed a significant association between the evidence of irreversible kidney damage (renal sclerosis, tubular atrophy, or interstitial fibrosis) with the negative impact on achieving remission.

We recently showed that histological findings in repeat kidney biopsies of LN patients commonly present discordance in relation to clinical expression. At repeat biopsy, chronicity index was more influential over CKD progression than the shift to lower pathological classes [27, 30]. Histological data from repeat kidney biopsies in LN could be useful to guide therapeutic approach [27]. For this reason, prospective randomized studies such as "Per-protocol repeat kidney biopsy in incident cases of LN" should shed some more light on the possibility of changing the course of lupus nephritis.

In the present study, the presence of thrombocytopenia below 50,000 cells/mm3 has been identified as an important risk factor for the progression of renal damage. The finding of thrombocytopaenia was not in the context of a manifestation associated with thrombotic microangiopathy, but as a more severe extrarenal systemic manifestation of systemic lupus erythematosus. Clark et al. observed that kinetic studies performed in patients with SLE have shown evidence of platelet consumption in the majority, and it is agreed by most authors that patients with SLE demonstrate evidence of compensated thrombocytolysis [31]. In the past this had been thought to relate to the presence of a circulating antibody to platelets [32, 33], but more recent evidence supports the hypothesis of the antiplatelet factor in SLE being a circulating immune complex [32, 34, 35]. Hence thrombocytopenia may reflect interaction of the platelet with an immune complex of critical size or configuration, which results in tissue damage and associated disease activity [36]. The presence of thrombocytopenia at the debut of SLE should alert us to a worse evolution of patients with lupus nephritis, and therefore we should try to be more forceful in our immunosuppressive treatment. Haematological abnormalities, especially thrombocytopenia, are highly prevalent among patients with systemic lupus erythematosus and at the same time it has been reported as a significant prognostic factor of SLE course [37]. Several studies have shown that the significance platelet count has a negative correlation with disease activity in SLE patients (arthritis, neurologic manifestations, and nephritis), whatever the associated manifestations, and it should be considered as a prognostic factor, identifying patients with aggressive disease course [36–38].

This study is subject to limitations due to the small sample size and its single-centre retrospective nature. However, strengths include that it is a real-world experience in standard clinical practice and a long follow-up time, giving homogeneity to our histological results. Our predictive model shows good discrimination capacity, with area under the curve close to 0.9.

Our study suggests that this prognosis index may be useful in clinical practice to detect which patients with lupus nephritis may have a worse renal prognosis and to modify our therapeutic approach to preserve kidney function. In order to stratify patients into different risk grades, future research is needed for internal and external validation with another cohort of patients.

In conclusion, we have developed a prognostic index of poor renal evolution in patients with LN that combines demographic, clinical, analytical and histopathological factors, easy to use in routine clinical practice and that could be an effective tool in the early detection and management of patients.

## Data Availability

Not applicable.

## Abbreviations

aB2GP1: Anti-B2 glycoprotein1
ACL: Anticardiolipin
ACR: American College of Rheumatology
AIC and BIC: Akaike and Bayesian information criteria
CKD: Chronic kidney disease
CKD-EPI: Chronic Kidney Disease Epidemiology Collaboration
eGFR: Glomerular filtration rate
ESKD: End-stage kidney disease
ISN/RNP: International Society of Nephrology/Renal Pathology Society
LA: Lupus anticoagulant
LN: Lupus nephritis
SCr: Serum creatinine
SLE: Systemic Lupus Erythematosus
SLEDAI: Systemic Lupus Erythematosus Disease Activity Index
SLICC: Systemic Lupus International Collaborating Centers
uPCR: Urine protein/creatinine rate
TMA: Thrombotic microangiopathy
